# The Selection of Hospital Staffs

**Published:** 1916-11-04

**Authors:** 


### The Hospital

The Workers' Newspaper of Administrative Medicine and Institutional Life, Administration, National Insurance and Health.

No. 1587, Vol. LXI. SATURDAY, NOVEMBER 4, 1916. PRICE ONE PENNY.

### THE SELECTION OF HOSPITAL STAFFS.

In another column (p. 85) we print this week a contribution to the question of the selection of resident and visiting staffs at hospitals, from the pen of Dr. S. S. Goldwater, himself a highly esteemed and successful New York superintendent. Many of Dr. Goldwater's principles have been enunciated at various times within the past few years in this Journal; but perhaps never before have they been laid down with such precision and such completeness, or supported by such cogent reasoning. The danger of specialising too early in a career, the too ready resort to exploratory operation when greater diagnostic care would avoid the necessity, the need for practical training of the surgeon in both up-to-date medicine and pathology, all these have been insisted on before, and represent tendencies which are just as visible on this side of the Atlantic as upon the other. The optimum duration of resident medical appointments is a large question which, as Dr. Goldwater indicates, has more than one side to it; it has not received due consideration in this country among hospital managers, and it is noticeable that even Dr. Goldwater is shy of formulating any hard-and-fast conclusions on the subject.

Passing from the pupillary or apprenticeship period of the young doctor, attention is next turned to the selection of the visiting staff; and by this the Mount Sinai superintendent appears to mean the surgical staff alone. Great emphasis is laid upon the need for reviewing all such appointments after an interval, in order to exclude those whose attainments have not come up to the standard expected when their appointments were made. It may be inferred that this principle has made no more headway in America than in Great Britain, perhaps even not so much. Certainly it is gradually, if slowly, gaining recognition in this country, and has long been advocated in this Journal. Nor need it be confined to the single probationary period cautiously suggested by Dr. Goldwater. The system of reviewing all visiting staff appointments quinquennially has much in its favour; and those institutions which have adopted such a regulation in this country seem to be very pleased with its practical working. Another matter in which American institutions evidently suffer much as British hospitals do is in connection with undue plurality of staff appointments. A man, whether he be surgeon or physician or what not, cannot do adequate justice to four or five separate hospitals, especially if he has a large private practice as well. On the other hand, to lay down a rule forbidding plurality altogether would probably not conduce to the highest standard of service to the sick in hospital: cases must be considered on their merits, a much easier task theoretically than it is in actual workaday practice.

Fortunately the precautions against "graft" and corruption over visiting staff appointments are much less necessary, in our judgment, in this country than they appear to be in the United States. But it would be foolish affectation to deny that considerations other than the intrinsic merit of each candidate's qualifications for office do sway elections to staff vacancies in British hospitals. Hospital managers will do well to lay to heart what Dr. Goldwater has to say on this topic.

The second half of Dr. Goldwater's paper, which we hope to publish very shortly, is equally full of wisdom on certain large questions which seem to have got further advanced towards a solution in the American hospital world than they have in this country. Discussing the enforcement of discipline on hospital surgeons—and the principle applies equally, of course, to physicians, gynæcologists, pathologists, and other specialists—he lays down the rule that when a member of the visiting staff is so preoccupied with private practice that he cannot do justice to his hospital cases he must be replaced by someone else who can. This simple and admirable principle is, however, as all hospital administrators know, exceedingly difficult to apply, because there is no clear test as to when a member of the visiting staff is or is not failing in his duty to his hospital patients. Dr. Goldwater mentions the system of appointing full-time salaried visiting surgeons to avoid this dilemma, though this system as a rule is adopted for another purpose—namely, the promotion of research and the careful teaching of students. Short of this system, which is costly, he mentions that the trustees of the most important municipal hospital in New York pay salaries to the chiefs of their medical and surgical services for part-time work, in this case for a minimum of four hours a day.

It would seem as if such a system is a stepping-stone to the payment of all members of visiting staffs. But the principle need not be carried to this, its logical conclusion; there is in America, though not to any extent in England, yet a third form of compensation to visiting staff surgeons besides direct money payment and indirect opportunities of acquiring fame and private practice. This third chance of recompense for the time devoted to the service of a hospital consists in the privilege of caring for paying patients in the private wards of the hospital. It is no small advantage to have at disposal well-equipped private wards in close proximity to the hospital wards in which the surgeon or physician spends so much of his working day, and to be able to command the resources of first-class laboratories, operating theatres, and other amenities of a first-rate hospital whenever they are required. It has long been maintained in these pages that in Great Britain we have not progressed nearly fast enough along the lines, which have proved so successful in America, of the private ward in the public hospital. With us the nursing home has monopolised the private patient. Without any desire to be unfair to nursing homes and their proprietors, many of whom do most admirable work, it must be admitted that the average nursing home is not such a good place to be ill in as the average private ward in a public hospital.

